# Elevated preoperative CA125 is associated with poor survival in patients with metastatic colorectal cancer undergoing primary tumor resection: a retrospective cohort study

**DOI:** 10.1093/gastro/goac020

**Published:** 2022-06-14

**Authors:** Jun-Hua Huang, Hua-Shan Liu, Tuo Hu, Zong-Jin Zhang, Xiao-Wen He, Tai-Wei Mo, Xiao-Feng Wen, Ping Lan, Lei Lian, Xian-Rui Wu

**Affiliations:** Department of Colorectal Surgery, The Sixth Affiliated Hospital of Sun Yat-sen University, Guangzhou, Guangdong, P. R. China; Guangdong Provincial Key Laboratory of Colorectal and Pelvic Floor Diseases, The Sixth Affiliated Hospital of Sun Yat-sen University, Guangzhou, Guangdong, P. R. China; Department of Colorectal Surgery, The Sixth Affiliated Hospital of Sun Yat-sen University, Guangzhou, Guangdong, P. R. China; Guangdong Provincial Key Laboratory of Colorectal and Pelvic Floor Diseases, The Sixth Affiliated Hospital of Sun Yat-sen University, Guangzhou, Guangdong, P. R. China; Department of Colorectal Surgery, The Sixth Affiliated Hospital of Sun Yat-sen University, Guangzhou, Guangdong, P. R. China; Guangdong Provincial Key Laboratory of Colorectal and Pelvic Floor Diseases, The Sixth Affiliated Hospital of Sun Yat-sen University, Guangzhou, Guangdong, P. R. China; Department of Colorectal Surgery, The Sixth Affiliated Hospital of Sun Yat-sen University, Guangzhou, Guangdong, P. R. China; Guangdong Provincial Key Laboratory of Colorectal and Pelvic Floor Diseases, The Sixth Affiliated Hospital of Sun Yat-sen University, Guangzhou, Guangdong, P. R. China; Department of Colorectal Surgery, The Sixth Affiliated Hospital of Sun Yat-sen University, Guangzhou, Guangdong, P. R. China; Guangdong Provincial Key Laboratory of Colorectal and Pelvic Floor Diseases, The Sixth Affiliated Hospital of Sun Yat-sen University, Guangzhou, Guangdong, P. R. China; Department of Colorectal Surgery, The Sixth Affiliated Hospital of Sun Yat-sen University, Guangzhou, Guangdong, P. R. China; Guangdong Provincial Key Laboratory of Colorectal and Pelvic Floor Diseases, The Sixth Affiliated Hospital of Sun Yat-sen University, Guangzhou, Guangdong, P. R. China; Department of Colorectal Surgery, The Sixth Affiliated Hospital of Sun Yat-sen University, Guangzhou, Guangdong, P. R. China; Guangdong Provincial Key Laboratory of Colorectal and Pelvic Floor Diseases, The Sixth Affiliated Hospital of Sun Yat-sen University, Guangzhou, Guangdong, P. R. China; Department of Colorectal Surgery, The Sixth Affiliated Hospital of Sun Yat-sen University, Guangzhou, Guangdong, P. R. China; Guangdong Provincial Key Laboratory of Colorectal and Pelvic Floor Diseases, The Sixth Affiliated Hospital of Sun Yat-sen University, Guangzhou, Guangdong, P. R. China; Guangdong Provincial Key Laboratory of Colorectal and Pelvic Floor Diseases, The Sixth Affiliated Hospital of Sun Yat-sen University, Guangzhou, Guangdong, P. R. China; Department of Gastric Surgery, The Sixth Affiliated Hospital of Sun Yat-sen University, Guangzhou, Guangdong, P. R. China; Department of Colorectal Surgery, The Sixth Affiliated Hospital of Sun Yat-sen University, Guangzhou, Guangdong, P. R. China; Guangdong Provincial Key Laboratory of Colorectal and Pelvic Floor Diseases, The Sixth Affiliated Hospital of Sun Yat-sen University, Guangzhou, Guangdong, P. R. China

**Keywords:** metastatic colorectal cancer, primary tumor resection, CA125, prognosis, survival

## Abstract

**Background:**

The impact of the preoperative carbohydrate antigen 125 (CA125) level on the survival of metastatic colorectal cancer (CRC) patients undergoing primary tumor resection (PTR) remains uncertain. The aim of this study was to assess the prognostic value in overall survival (OS) and cancer-specific survival (CSS) between patients with and without an elevated preoperative CA125 level.

**Methods:**

All metastatic CRC patients receiving PTR between 2007 and 2017 at the Sixth Affiliated Hospital of Sun Yat-sen University (Guangzhou, China) were retrospectively included. OS and CSS rates were compared between patients with and without elevated preoperative CA125 levels.

**Results:**

Among 326 patients examined, 46 (14.1%) exhibited elevated preoperative CA125 levels and the remaining 280 (85.9%) had normal preoperative CA125 levels. Patients with elevated preoperative CA125 levels had lower body mass index, lower preoperative albumin level, lower proportion of preoperative chemotherapy, higher carcinoembryonic antigen and carbohydrate antigen 19–9 (CA19–9) levels, poorer differentiation, and more malignant histopathological type than patients with normal preoperative CA125 levels. In addition, patients with elevated preoperative CA125 levels exhibited more advanced pathological T and N stages, more peritoneal metastasis, and more vessel invasion than patients with normal preoperative CA125 levels. Moreover, the primary tumor was more likely to be located at the colon rather than at the rectum in patients with elevated CA125 levels. Both OS and CSS rates in patients with elevated preoperative CA125 levels were significantly lower than those in patients with normal preoperative CA125 levels. Multivariate Cox regression analysis revealed that an elevated preoperative CA125 level was significantly associated with poor prognosis in metastatic CRC patients undergoing PTR. The hazard ratio (HR) in OS was 2.36 (95% confidence interval [CI], 1.67–3.33, *P* < 0.001) and the HR in CSS was 2.50 (95% CI, 1.77–3.55, *P* < 0.001). The survival analysis stratified by peritoneal metastasis also demonstrated that patients with elevated preoperative CA125 levels had lower OS and CSS rates regardless of peritoneal metastasis.

**Conclusion:**

Based on an analysis of metastatic CRC patients undergoing PTR, an elevated preoperative CA125 level was associated with poor prognosis, which should be taken into consideration in clinical practice.

## Introduction

The incidence of colorectal cancer (CRC) ranks third among all cancer types worldwide and it is the second leading cause of malignancy-related mortality [1, 2]. Approximately 20% of patients diagnosed with CRC present with synchronous metastatic diseases [3] and 75%–90% of these cases are unresectable [4, 5]. According to previous studies, a considerable proportion of CRC patients with unresectable synchronous metastatic diseases have poor prognosis [6, 7]. Primary tumor resection (PTR) is one of the therapeutic options for these patients, which can relieve tumor-related complications and prevent life-threatening conditions such as intractable bleeding, intestinal obstruction, and perforation [8]. Although some studies have revealed the survival benefits of PTR for asymptomatic metastatic CRC patients [9–11], others have reported the opposite results [12, 13]. Therefore, studies evaluating the clinicopathological factors associated with the prognosis in metastatic CRC patients undergoing PTR are warranted.

A certain number of risk factors related to prognosis for metastatic CRC patients receiving PTR were proposed, such as age, preoperative carcinoembryonic antigen (CEA) level, tumor site, TNM stage, tissue differentiation, and histopathology [14–19]. Carbohydrate antigen 125 (CA125) was first utilized as a sensitive tumor biomarker in ovarian cancer [20, 21]. Afterwards, CA125 was found in gastrointestinal tumor cells [22] and served as a tumor biomarker in the routine testing of patients with gastrointestinal tumors, playing an important role in tumorigenesis, tumor proliferation, and metastasis [21, 23, 24]. Moreover, CA125 is reported as a sensitive predictor of peritoneal dissemination and exhibits a prognostic value for recurrence and survival in CRC patients [25–28]. However, the association between preoperative CA125 levels and prognosis for metastatic CRC patients receiving PTR remains uncertain.

The aim of this study was to assess the impact of elevated preoperative CA125 levels on the survival of metastatic CRC patients receiving PTR.

## Patients and methods

### Patients

Patients’ medical data were collected and sorted by physician assistants. Detailed information was then registered by the staff using the colorectal cancer database of the Sixth Affiliated Hospital of Sun Yat-sen University (Guangzhou, China) after informed consent. In addition, subsequent regular follow-ups were arranged via phone, mail, and/or hospital visits whenever possible to update the information of our database. In this study, patients with CRC undergoing PTR at the Sixth Affiliated Hospital of Sun Yat-sen University between January 2007 and December 2017 were included. Demographic variables, preoperative symptoms, imaging data, biochemical tests, post-operative pathological status, and follow-up data were collected from the colorectal cancer database of our institution. Electronic and paper medical records were carefully reviewed when necessary. This study was conducted in accordance with the 1964 Helsinki Declaration and its subsequent amendments. This study was approved by the Institutional Review Board of the Sixth Affiliated Hospital of Sun Yat-sen University. This is a retrospective study with demonstrated minimal risk and we petition for the waiver of informed consent. This work has been reported in line with the STROCSS criteria [29].

### Patient groups

Patient groups were defined according to the clinical cut-off value (35 U/mL) of the preoperative CA125 level. Patients with a preoperative CA125 of >35 U/mL were assigned to the “elevated preoperative CA125” group, while those with a preoperative of CA125 ≤35 U/mL were classified into the “normal preoperative CA125” group. The test of CA125 for all patients was performed within 1 month before surgery.

### Definition of variables

Demographic and clinicopathological variables were defined and analysed as follows: gender (female vs male), age, body mass index (BMI), concurrent co-morbidity (other diseases in addition to CRC, such as cardiovascular, respiratory, metabolic, non-neoplastic digestive, and urinary diseases), hypoalbuminemia (preoperative albumin value of <35 g/L), anemia (preoperative hemoglobin value of <120 and <110 g/L for males and females, respectively), elevated preoperative CEA (preoperative CEA level of >5 ng/mL), elevated preoperative carbohydrate antigen 19–9 (CA19–9) (preoperative CA19–9 level of >37 U/mL), clinical T stage (cT4 vs cT1 to cT3), clinical N stage (cN+ vs cN0), tumor site (the rectum vs the colon), differentiation (poor vs well; well, high, and moderate differentiation; poor, poor differentiation, and undifferentiation), histopathology (mucinous adenocarcinoma and signet-ring cell carcinoma vs adenocarcinoma), liver metastasis, lung metastasis, peritoneal metastasis, multiple metastasis (metastatic lesions involve two or more organs), pathological T stage (pT4 vs pT1 to pT3), pathological N stage (pN+ vs pN0), vessel invasion, nerve invasion, gene mutation (including *KRAS*, *NRAS*, *BRAF*, and *PIK3CA* mutations), microsatellite instability (MSI) (MSI-H vs MSI-L), post-operative complication (Grade III or higher complications according to Dindo-Clavien Classifications of surgical complications [30]), preoperative chemotherapy, and post-operative chemotherapy.

### Outcomes

The primary outcome of this study was overall survival (OS), which was calculated from the date of PTR treatment to the date of death from any causes or the last follow-up. The secondary outcome was cancer-specific survival (CSS), which was calculated from the date of PTR treatment to the date of death from CRC or the last follow-up.

### Inclusion and exclusion criteria

Patients included in this study must meet all the following inclusion criteria: (i) patients diagnosed with CRC; (ii) patients with synchronous metastatic lesions; (iii) patients without emergent tumor-related complications such as intractable bleeding, intestinal obstruction, and perforation; (iv) patients receiving PTR; and (v) patients with regular follow-up up to the time of 1 January 2021 at our institution. The exclusion criteria were as follows: (i) patients with recurrent CRC; (ii) patients with multiple primary carcinomas; (iii) patients with resectable or potentially resectable metastatic lesions; and (iv) patients with missing data exceeding 20% of all clinicopathological variables.

### Statistical analysis

Statistical analysis was conducted using the statistical package for social sciences (SPSS version 26.0.0.0, IBM SPSS statistics) and R software (version 4.0.3; https://www.Rproject.org). Descriptive statistics were computed for all variables. These included means and standard deviations or medians and interquartile ranges for continuous factors and frequencies for categorical factors. Patients’ clinicopathological characteristics were compared between the elevated preoperative CA125 and normal preoperative CA125 groups. Continuous variables were analysed by using the two-sided Student *t*-test and categorical variables were analysed by using the chi-square test (or the Fisher exact test if necessary). OS and CSS were estimated using the Kaplan–Meier analysis and compared using the log-rank test between elevated preoperative CA125 and normal preoperative CA125 groups and between the subgroups stratified by peritoneal metastasis. Both univariate and multivariate analyses were performed using the Cox’s proportional hazards regression model. The multivariate analysis was performed using the enter method with entry criterion as *P* < 0.05 and removal criterion as *P* *>* 0.10. A *P*-value of <0.05 was considered statistically significant in this study.

## Results

### Patient characteristics

A total of 326 eligible patients were evaluated, including 46 (14.1%) with elevated preoperative CA125 levels. Patients with elevated preoperative CA125 levels had a significantly lower mean BMI value than those with normal preoperative CA125 levels (21.2 ± 2.8 vs 22.2 ± 3.1 kg/m^2^, *P* *=* 0.024) (Table 1) and they were more likely to have hypoalbuminemia (37.0% vs 7.9%, *P* < 0.001), high preoperative CEA levels (76.1% vs 57.9%, *P* *=* 0.019), high preoperative CA19-9 levels (50.0% vs 30.7%, *P* *=* 0.01), advanced stage of clinical T stage (45.7% vs 27.5% at T4 stage, *P* *=* 0.013), poor differentiation (56.5% vs 15.7%, *P* < 0.001), malignant histopathological type (21.7% vs 9.3%, *P* *=* 0.013), peritoneal metastasis (54.3% vs 10.7%, *P* < 0.001), advanced stage of pathological T stage (41.3% vs 14.3% at T4 stage, *P* < 0.001), advanced stage of pathological N stage (87.0% vs 68.9% at N+ stage, *P* *=* 0.012), and vessel invasion (39.1% vs 15.7%, *P* < 0.001). The primary tumor was more likely to be located at the colon in metastatic CRC patients with elevated preoperative CA125 levels than in those with normal preoperative CA125 levels (71.7% vs 44.6%, *P* *=* 0.001). The proportion of patients receiving preoperative chemotherapy was higher in patients with elevated preoperative CA125 levels than in those with normal preoperative CA125 levels (15.2% vs 36.4%, *P* *=* 0.005). There was no significant difference in other clinicopathological characteristics between elevated preoperative CA125 and normal preoperative CA125 groups (*P* *>* 0.05) (Table 1).

**Table 1.** Characteristics of 326 patients with metastatic colorectal cancer undergoing primary tumor resection

Characteristic	All cases	Elevated preoperative CA125	Normal preoperative CA125	*P-*value
Number of patients	326	46 (14.1)	280 (85.9)	
Gender				0.687
Male	221 (67.8)	30 (65.2)	191 (68.2)	
Female	105 (32.2)	16 (34.8)	89 (31.8)	
Age,^a^ years	59.2 ± 13.6	60.9 ± 15.2	59.0 ± 13.3	0.421
Body mass index,^a^ kg/m^2^	22.1 ± 3.1	21.2 ± 2.8	22.2 ± 3.1	0.024
Concurrent co-morbidity	117 (35.9)	16 (34.8)	101 (36.1)	0.866
Hypoalbuminemia	39 (12.0)	17 (37.0)	22 (7.9)	<0.001
Anemia	135 (41.4)	24 (52.2)	111 (39.6)	0.11
Elevated preoperative CEA (>5 ng/mL)	197 (60.4)	35 (76.1)	162 (57.9)	0.019
Elevated preoperative CA19-9 (>37 U/mL)	109 (33.4)	23 (50.0)	86 (30.7)	0.01
Clinical T stage				0.013
cT1 to cT3	228 (69.9)	25 (54.3)	203 (72.5)	
cT4	98 (30.1)	21 (45.7)	77 (27.5)	
Clinical N stage				0.23
cN0	83 (25.5)	15 (32.6)	68 (24.3)	
cN+	243 (74.5)	31 (67.4)	212 (75.7)	
Tumor site				0.001
Colon	158 (48.5)	33 (71.7)	125 (44.6)	
Rectum	168 (51.5)	13 (28.3)	155 (55.4)	
Differentiation				<0.001
Well	256 (78.5)	20 (43.5)	236 (84.3)	
Poor	70 (21.5)	26 (56.5)	44 (15.7)	
Histopathology				0.013
Adenocarcinoma	290 (89.0)	36 (78.3)	254 (90.7)	
Mucinous adenocarcinoma and signet-ring cell carcinoma	36 (11.0)	10 (21.7)	26 (9.3)	
Liver metastasis	231 (70.9)	28 (60.9)	203 (72.5)	0.108
Lung metastasis	117 (35.9)	11 (23.9)	106 (37.9)	0.068
Peritoneal metastasis	55 (16.9)	25 (54.3)	30 (10.7)	<0.001
Multiple metastasis	112 (34.4)	21 (45.7)	91 (32.5)	0.082
Pathological T stage				<0.001
pT1 to pT3	267 (81.9)	27 (58.7)	240 (85.7)	
pT4	59 (18.1)	19 (41.3)	40 (14.3)	
Pathological N stage				0.012
pN0	93 (28.5)	6 (13.0)	87 (31.1)	
pN+	233 (71.5)	40 (87.0)	193 (68.9)	
Vessel invasion	62 (19.0)	18 (39.1)	44 (15.7)	<0.001
Nerve invasion	94 (28.8)	16 (34.8)	78 (27.9)	0.337
Gene mutation	114 (40.3)	21 (48.8)	93 (38.8)	0.214
MSI				0.999
MSI-L	261 (94.2)	36 (94.7)	225 (94.1)	
MSI-H	16 (5.8)	2 (5.3)	14 (5.9)	
Post-operative complication	24 (7.4)	6 (13.0)	18 (6.4)	0.111
Preoperative chemotherapy	109 (33.4)	7 (15.2)	102 (36.4)	0.005
Post-operative chemotherapy	222 (68.1)	27 (58.7)	195 (69.6)	0.14

a The values of the two items are presented as mean ± standard deviation; the values of other items are presented as the number of patients followed by percentages in parentheses.

### Impact of elevated preoperative CA125 levels on OS in metastatic CRC patients undergoing PTR

**Figure 1.**
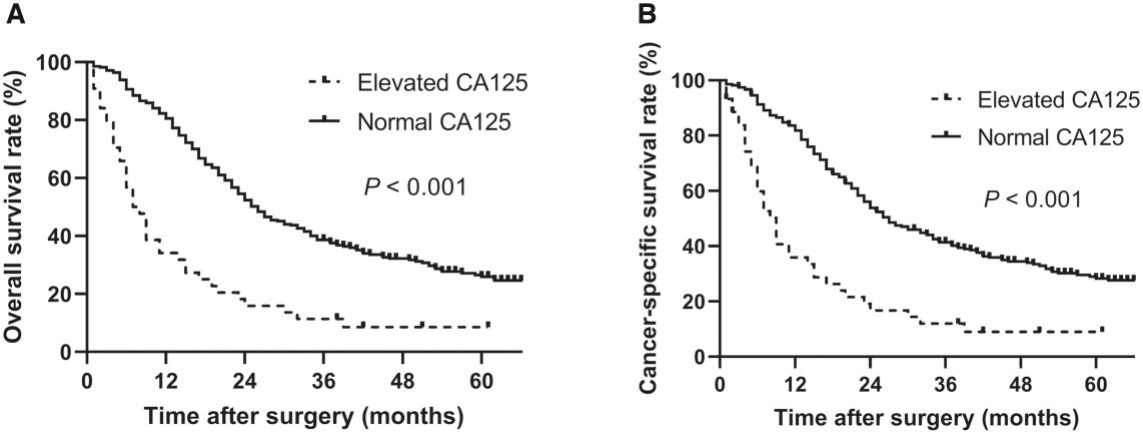
Kaplan–Meier curves for overall survival (A) and cancer-specific survival (B) in patients with and without elevated preoperative CA125 levels

The percentage of metastatic CRC patients after PTR who were lost to follow-up was 21.6%. The results showed OS was significantly lower in patients with elevated preoperative CA125 levels than in those with normal preoperative CA125 levels (*P* < 0.001) (Figure 1A). The median OS time was 7.0 months (95% confidence interval [CI], 4.8–9.2 months) for patients with elevated preoperative CA125 levels and 25.0 months (95% CI, 21.9–28.2 months) for those with normal preoperative CA125 levels. Univariate Cox regression analysis showed that an elevated preoperative CA125 level was significantly associated with poor prognosis, with a hazard ratio (HR) of 2.95 (95% CI, 2.10–4.13, *P* < 0.001) (Table 2). With regard to other clinicopathological variables, age (*P* < 0.001), hypoalbuminemia (*P* *=* 0.002), anemia (*P* *=* 0.036), elevated preoperative CEA (*P* < 0.001), elevated preoperative CA19-9 (*P* < 0.001), clinical T stage (*P* < 0.001), differentiation (*P* < 0.001), histopathology (*P* *=* 0.037), peritoneal metastasis (*P* < 0.001), multiple metastasis (*P* < 0.001), pathological T stage (*P* *=* 0.001), pathological N stage (*P* *=* 0.003), post-operative complication (*P* *=* 0.012), and post-operative chemotherapy (*P* < 0.001) were all associated with the prognosis in metastatic CRC patients receiving PTR (Table 2). The association between an elevated preoperative CA125 level and poor prognosis was further confirmed by multivariate analysis (HR, 2.36; 95% CI, 1.67–3.33, *P* < 0.001) after adjusting with anemia (HR, 1.35; 95% CI, 1.04–1.75, *P* *=* 0.026), elevated preoperative CEA (HR, 1.50; 95% CI, 1.14–1.97, *P* *=* 0.004), clinical T stage (HR, 1.43; 95% CI, 1.09–1.88, *P* *=* 0.01), multiple metastasis (HR, 1.62; 95% CI, 1.25–2.11, *P* < 0.001), post-operative complication (HR, 1.91; 95% CI, 1.19–3.07, *P* *=* 0.008), and post-operative chemotherapy (HR, 0.50; 95% CI, 0.38–0.65, *P* < 0.001) (Table 3).

**Table 2.** Univariate analysis of risk factors associated with prognosis for overall survival in metastatic colorectal cancer patients undergoing primary tumor resection

Characteristic	Overall survival		
	Hazard ratio	95% confidence interval	*P*-value
Gender (female vs male)	0.92	0.71–1.20	0.54
Age, every 1-year increase	1.02	1.01–1.03	<0.001
BMI, every 1-kg/m^2^ increase	0.97	0.93–1.01	0.092
Concurrent co-morbidity (yes vs no)	1.23	0.95–1.59	0.109
Hypoalbuminemia (yes vs no)	1.74	1.22–2.49	0.002
Anemia (yes vs no)	1.31	1.02–1.68	0.036
Elevated preoperative CEA (>5 ng/mL) (yes vs no)	1.79	1.38–2.33	<0.001
Elevated preoperative CA199 (>37 U/mL) (yes vs no)	1.67	1.29–2.16	<0.001
Elevated preoperative CA125 (>35 U/mL) (yes vs no)	2.95	2.10–4.13	<0.001
Clinical T stage (cT4 vs cT1 to cT3)	1.63	1.26–2.13	<0.001
Clinical N stage (cN+ vs cN0)	1.22	0.91–1.63	0.187
Tumor site (rectum vs colon)	1.06	0.82–1.35	0.668
Differentiation (poor vs well)	1.77	1.32–2.36	<0.001
Histopathology (mucinous adenocarcinoma and signet-ring cell carcinoma vs adenocarcinoma)	1.49	1.02–2.16	0.037
Liver metastasis (yes vs no)	1.06	0.81–1.39	0.689
Lung metastasis (yes vs no)	0.80	0.61–1.03	0.086
Peritoneal metastasis (yes vs no)	2.00	1.47–2.74	<0.001
Multiple metastasis (yes vs no)	1.76	1.36–2.27	<0.001
Pathological T stage (pT4 vs pT1 to pT3)	1.70	1.24–2.32	0.001
Pathological N stage (pN+ vs pN0)	1.54	1.16–2.05	0.003
Vessel invasion (yes vs no)	1.29	0.94–1.75	0.111
Nerve invasion (yes vs no)	1.18	0.90–1.55	0.128
Gene mutation (yes vs no)	1.23	0.94–1.61	0.13
MSI (MSI-H vs MSI-L)	0.75	0.42–1.35	0.342
Post-operative complication (yes vs no)	1.80	1.14–2.84	0.012
Preoperative chemotherapy (yes vs no)	0.86	0.66–1.12	0.261
Post-operative chemotherapy (yes vs no)	0.45	0.35–0.58	<0.001

**Table 3.** Multivariate analysis of risk factors associated with prognosis for overall survival in metastatic colorectal cancer patients undergoing primary tumor resection

Characteristic	Overall survival		
	Hazard ratio	95% confidence interval	*P*-value
Age, every 1-year increase	1.01	0.99–1.02	0.212
Hypoalbuminemia (yes vs no)	1.18	0.76–1.81	0.465
Anemia (yes vs no)	1.35	1.04–1.75	0.026
Elevated preoperative CEA (>5 ng/mL) (yes vs no)	1.50	1.14–1.97	0.004
Elevated preoperative CA199 (>37 U/mL) (yes vs no)	1.22	0.91–1.63	0.18
Elevated preoperative CA125 (>35 U/mL) (yes vs no)	2.36	1.67–3.33	<0.001
Clinical T stage (cT4 vs cT1 to cT3)	1.43	1.09–1.88	0.01
Differentiation (poor vs well)	0.97	0.92–1.52	0.161
Histopathology (mucinous adenocarcinoma and signet-ring cell carcinoma vs adenocarcinoma)	1.22	0.69–2.15	0.49
Peritoneal metastasis (yes vs no)	1.20	0.82–1.76	0.34
Multiple metastasis (yes vs no)	1.62	1.25–2.11	<0.001
Pathological T stage (pT4 vs pT1 to pT3)	1.23	0.85–1.78	0.265
Pathological N stage (pN+ vs pN0)	1.34	0.98–1.83	0.066
Post-operative complication (yes vs no)	1.91	1.19–3.07	0.008
Post-operative chemotherapy (yes vs no)	0.50	0.38–0.65	<0.001

### Impact of elevated preoperative CA125 levels on CSS in metastatic CRC patients undergoing PTR

A similar statistical difference in CSS between elevated and normal preoperative CA125 groups was obtained (log-rank test, *P* < 0.001) (Figure 1B). The median CSS time for patients with elevated preoperative CA125 levels was 8.0 months (95% CI, 5.6–10.4 months), whereas the median CSS time for those with normal preoperative CA125 levels was 27.0 months (95% CI, 22.2–31.8 months). Univariate Cox regression analysis showed that an elevated preoperative CA125 level was significantly associated with short CSS (HR, 3.05; 95% CI, 2.16–4.32, *P* < 0.001) (Table 4). Other clinicopathological variables, such as age (*P* *=* 0.005), hypoalbuminemia (*P* *=* 0.019), elevated preoperative CEA (*P* < 0.001), elevated preoperative CA19-9 (*P* < 0.001), clinical T stage (*P* *=* 0.001), differentiation (*P* < 0.001), histopathology (*P* *=* 0.023), peritoneal metastasis (*P* < 0.001), multiple metastasis (*P* < 0.001), pathological T stage (*P* *=* 0.002), pathological N stage (*P* < 0.001), and post-operative chemotherapy (*P* < 0.001) were significantly associated with prognosis (Table 4). The association between an elevated preoperative CA125 level and poor prognosis was further verified by multivariate analysis (HR, 2.50; 95% CI, 1.77–3.55, *P* < 0.001) after adjusting by clinical T stage (HR, 1.44; 95% CI, 1.09–1.90, *P* *=* 0.10), multiple metastasis (HR, 1.71; 95% CI, 1.31–2.22, *P* < 0.001), pathological N stage (HR, 1.59; 95% CI, 1.17–2.16, *P* *=* 0.003), and post-operative chemotherapy (HR, 0.48; 95% CI, 0.37–0.63, *P* < 0.001) (Table 5).

**Table 4.** Univariate analysis of risk factors associated with prognosis for cancer-specific survival in metastatic colorectal cancer patients undergoing primary tumor resection

Characteristic	Cancer-specific survival		
	Hazard ratio	95% confidence interval	*P*-value
Gender (female vs male)	0.95	0.72–1.24	0.697
Age, every 1-year increase	1.01	1.00–1.03	0.005
BMI, every 1-kg/m^2^ increase	0.97	0.94–1.01	0.206
Concurrent co-morbidity (yes vs no)	1.17	0.90–1.53	0.235
Hypoalbuminemia (yes vs no)	1.58	1.08–2.32	0.019
Anemia (yes vs no)	1.29	1.00–1.67	0.051
Elevated preoperative CEA (>5 ng/mL) (yes vs no)	1.80	1.37–2.35	<0.001
Elevated preoperative CA19-9 (>37 U/mL) (yes vs no)	1.69	1.30–2.20	<0.001
Elevated preoperative CA125 (>35 U/mL) (yes vs no)	3.05	2.16–4.32	<0.001
Clinical T stage (cT4 vs cT1 to cT3)	1.60	1.21–2.10	0.001
Clinical N stage (cN+ vs cN0)	1.35	0.99–1.84	0.062
Tumor site (rectum vs colon)	1.08	0.83–1.39	0.575
Differentiation (poor vs well)	1.82	1.35–2.45	<0.001
Histopathology (mucinous adenocarcinoma and signet-ring cell carcinoma vs adenocarcinoma)	1.55	1.06–2.27	0.023
Liver metastasis (yes vs no)	1.06	0.80–1.41	0.682
Lung metastasis (yes vs no)	0.81	0.62–1.06	0.121
Peritoneal metastasis (yes vs no)	1.98	1.43–2.73	<0.001
Multiple metastasis (yes vs no)	1.85	1.42–2.40	<0.001
Pathological T stage (pT4 vs pT1 to pT3)	1.67	1.21–2.31	0.002
Pathological N stage (pN+ vs pN0)	1.72	1.27–2.32	<0.001
Vessel invasion (yes vs no)	1.30	0.95–1.79	0.105
Nerve invasion (yes vs no)	1.25	0.95–1.65	0.113
Gene mutation (yes vs no)	1.24	0.94–1.63	0.137
MSI (MSI-H vs MSI-L)	0.81	0.45–1.45	0.475
Post-operative complication (yes vs no)	1.54	0.92–2.56	0.098
Preoperative chemotherapy (yes vs no)	0.89	0.67–1.16	0.376
Post-operative chemotherapy (yes vs no)	0.46	0.35–0.60	<0.001

**Table 5.** Multivariate analysis of risk factors associated with prognosis for cancer-specific survival in metastatic colorectal cancer patients undergoing primary tumor resection

Characteristic	Cancer-specific survival		
	Hazard ratio	95% confidence interval	*P*-value
Age, every 1-year increase	1.01	0.99–1.02	0.315
Hypoalbuminemia (yes vs no)	1.25	0.82–1.92	0.305
Elevated preoperative CEA (>5 ng/mL) (yes vs no)	1.26	0.93–1.72	0.141
Elevated preoperative CA19-9 (>37 U/mL) (yes vs no)	1.19	0.88–1.60	0.261
Elevated preoperative CA125 (>35 U/mL) (yes vs no)	2.50	1.77–3.55	<0.001
Clinical T stage (cT4 vs cT1 to cT3)	1.44	1.09–1.90	0.01
Differentiation (poor vs well)	0.93	0.58–1.48	0.75
Histopathology (mucinous adenocarcinoma and signet-ring cell carcinoma vs adenocarcinoma)	1.39	0.77–2.50	0.276
Peritoneal metastasis (yes vs no)	1.13	0.76–1.68	0.541
Multiple metastasis (yes vs no)	1.71	1.31–2.22	<0.001
Pathological T stage (pT4 vs pT1 to pT3)	1.15	0.78–1.69	0.476
Pathological N stage (pN+ vs pN0)	1.59	1.17–2.16	0.003
Post-operative chemotherapy (yes vs no)	0.48	0.37–0.63	<0.001

### Survival outcomes stratified by peritoneal metastasis in metastatic CRC patients undergoing PTR

**Figure 2.**
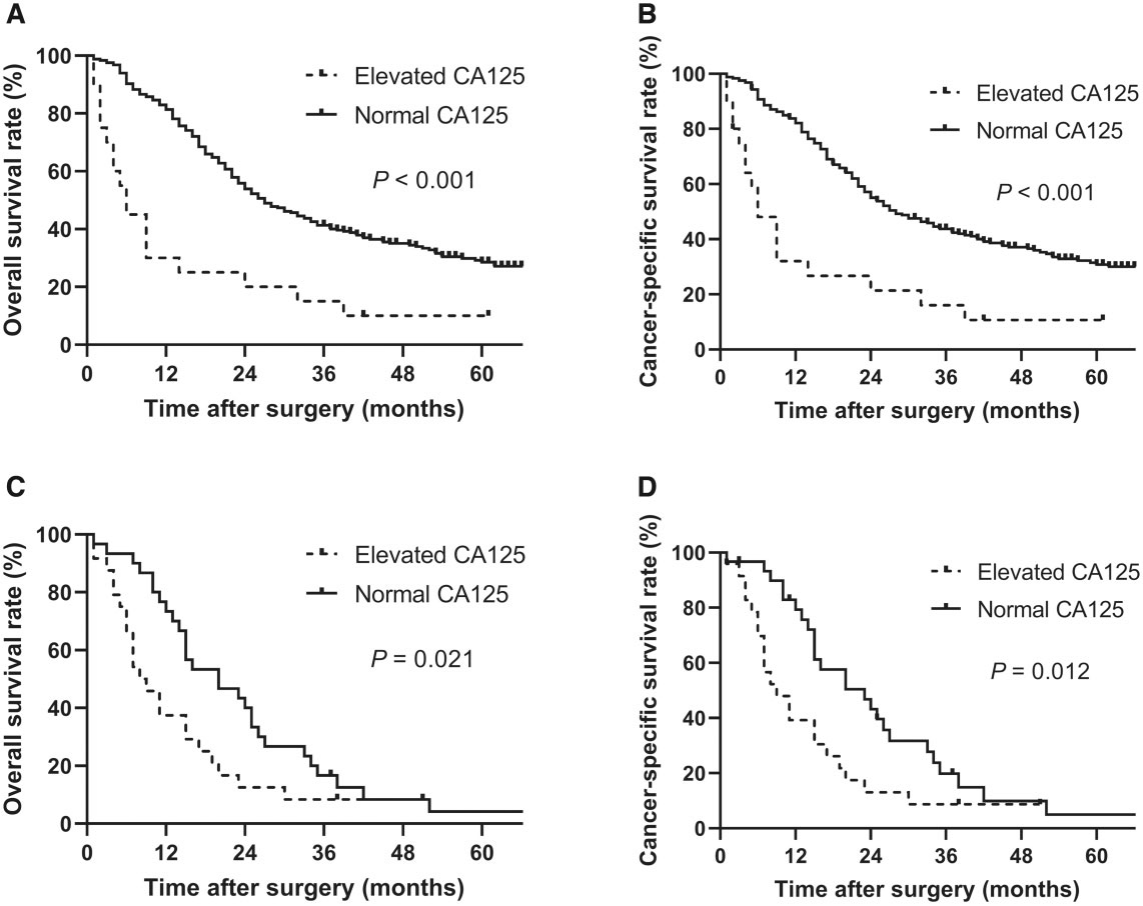
Kaplan–Meier curves for overall and cancer-specific survivals in patients with and without elevated preoperative CA125 levels stratified by peritoneal metastasis. (A) Overall survival of patients without peritoneal metastasis. (B) Cancer-specific survival of patients without peritoneal metastasis. (C) Overall survival of patients with peritoneal metastasis. (D) Cancer-specific survival of patients with peritoneal metastasis.

Survival analysis stratified by peritoneal metastasis was performed using Kaplan–Meier analysis. In metastatic CRC patients without peritoneal metastasis, both OS and CSS were significantly shorter in elevated preoperative CA125 patients than in normal preoperative CA125 patients (log-rank test, *P* < 0.001) (Figure 2A and B). The median OS was 6.0 months (95% CI, 3.0–9.0 months) and 26.0 months (95% CI, 21.0–31.0 months) for elevated preoperative CA125 and normal preoperative CA125 patients, respectively. The median CSS time was 6.0 months (95% CI, 2.6–9.4 months) and 28.0 months (95% CI, 22.5–33.5 months) for elevated preoperative CA125 and normal preoperative CA125 patients, respectively. In patients with peritoneal metastasis, the survival was also significantly different between two groups (OS, log-rank test *P* *=* 0.021; CSS, log-rank test *P* *=* 0.012) (Figure 2C and D). The median OS time was 8.0 months (95% CI, 5.1–11.0 months) and 20.0 months (95% CI, 9.3–30.7 months) for elevated preoperative CA125 and normal preoperative CA125 patients. As for CSS, the median survival time was 9.0 months (95% CI, 5.6–12.4 months) for elevated preoperative CA125 patients and 23.0 months (95% CI, 22.5–33.5 months) for normal preoperative CA125 patients.

## Discussion

In the present study, 46 of 326 patients (14.1%) exhibited elevated preoperative CA125 levels. As expected, elevated preoperative CA125 patients were more likely to have elevated preoperative CEA and CA19-9 levels, advanced T and N stages, poor differentiation type, malignant histopathological type, and peritoneal metastasis than normal preoperative CA125 patients. The major finding of this study was that an elevated preoperative CA125 level was significantly associated with short OS and CSS of metastatic CRC patients receiving PTR; patients with elevated preoperative CA125 levels had shorter median survival time than those with normal preoperative CA125 levels. In order to evaluate whether the impact of the CA125 level on survival was influenced by peritoneal metastasis, we compared the OS and CSS between these two groups using subgroup analysis stratified by peritoneal metastasis. Similar survival outcomes were obtained in patients both with and without peritoneal metastasis. The multivariate analysis demonstrated that the HR value of the elevated preoperative CA125 level was the highest among all risk factors for OS and CSS, which suggested that CA125 was an independent risk factor for the survival of patients with metastatic CRC following PTR.

CEA is a glycoprotein that is highly expressed in gastrointestinal cancer cells [31]. CEA was reported to enhance cancer invasion and metastasis by targeting intercellular adhesion molecules and promoting cellular aggregation [32]. Therefore, it is widely used as a tumor biomarker for gastrointestinal adenocarcinomas including CRC. Several studies have suggested that CEA is a prognostic predictor for CRC patients; however, whether its predictive efficiency is better than CA125 remains controversial [27, 28, 33]. In our study, we found that an elevated preoperative CEA level was an independent risk factor for OS, but not for CSS, in metastasis CRC patients receiving PTR, which suggested that the preoperative CA125 level might be more sensitive than preoperative CEA in prognosis for these patients.

The TNM stage was a well-known risk factor for the survival outcomes of CRC patients. Since the 8th edition of the TNM Classification of Malignant Tumors categorizes CRC with peritoneal metastasis as M1c, separated from M1a and M1b (metastasis to one and more than one organ, respectively), the poor prognosis for CRC patients with peritoneal metastasis has been widely accepted [34]. In accordance with this, patients with advanced T stage (T4) and positive N stage (N+) metastatic CRC were found to have poorer survival outcomes than those with T1 to T3 and negative N stage (N0) metastatic CRC in this study. In addition, multiple metastasis and peritoneal metastasis were also negative prognostic factors. Interestingly, in multivariate analysis of OS and CSS, multiple metastasis exhibited statistical significance while peritoneal metastasis did not, possibly because multiple metastasis referred to the involvement of two or more organs regardless of peritoneal metastasis in our design, and metastatic CRC patients with peritoneum-only metastasis might have a better prognosis than those with non-peritoneal multiple metastasis after receiving PTR.

Anemia and post-operative complications were independent prognostic risk factors for OS in CRC patients in this study. However, they did not meet statistical significance in CSS, possibly because these two factors, especially post-operative complications that referred to sever complications, might contribute to death due to reasons other than metastatic CRC. Post-operative chemotherapy was revealed to have a positive influence on patients’ survival and was an independent prognostic factor for OS and CSS. Consistently with our analysis, previous research has demonstrated that stage IV CRC patients treated with adjuvant chemotherapy obtained improved survival [35].

The present study identified CA125 as an independent risk factor associated with survival in metastatic CRC patients receiving PTR. Meanwhile, peritoneal metastasis in metastatic CRC patients might not be an absolute barrier for PTR. These findings are extremely valuable for the therapeutic option selection in the management of metastatic CRC patients. According to previous studies of metastatic CRC patients, researchers have focused more on the comparison of patients’ prognosis between surgery groups and chemotherapy-only groups [4, 8, 36, 37], with the fact that more and more metastatic CRC patients without emergent tumor-related complications received systemic therapy only [4, 12, 13]. However, in clinical practice, PTR might benefit patients with metastatic CRC in terms of survival [9–11]. Based on the controversial opinion, more studies should be performed to find out what kind of metastatic CRC patients can benefit from PTR management. In this study, a large cohort of metastatic CRC patients receiving PTR was included and analysed. Instead of concentrating on whether PTR had a prognostic benefit or not, we focused on selecting risk factors that affected prognosis after PTR. Moreover, compared to other research which demonstrated that CA125 had predictive value in tumor recurrence, peritoneal dissemination, and survival of non-metastatic CRC patients [25–28, 38–40], this study discovered that the preoperative CA125 level was an independent risk factor associated with the prognosis for metastatic CRC patients with PTR management.

There are some limitations in our study. First, it is a retrospective study that has its inherent shortages. In addition, since all patients in this study were collected from a single institution, the data of this study should be carefully interpreted. Future multicenter studies with large sample sizes are needed to further verify the prognostic value of CA125 in these patients.

In conclusion, for metastatic CRC patients receiving PTR, patients with elevated preoperative CA125 levels were found to have a poorer prognosis after surgery than those with normal preoperative CA125 levels. This information should be taken into consideration when metastatic CRC patients were consulted for PTR.

## Authors’ Contributions

J.H.H. contributed to conception and design of the study, acquisition of data, analysis and interpretation of data, and drafting of the manuscript. H.S.L. and T.H. contributed to analysis and interpretation of data and manuscript review. Z.J.Z. and X.W.H. contributed to interpretation of data and manuscript review. T.W.M. and X.F.W. contributed to acquisition of data and analysis. P.L., L.L., and X.R.W. contributed to conception and design of the study, analysis and interpretation of data, and critical revision of the manuscript for important intellectual content. All authors read and approved the final manuscript.

## Funding

This work was supported by the National Key R&D Program of China [no. 2017YFC1308800], the National Natural Science Foundation of China [no. 81870383], the Clinical Innovation Research Program of Bioland Laboratory (Guangzhou Regenerative Medicine and Health Guangdong Laboratory) [no. 2018GZR0201005], and the Science and Technology Planning Project of Guangzhou City [no. 201804010014].

## Conflict of Interest

None declared.
